# Human Dorsal Root Ganglia

**DOI:** 10.3389/fncel.2019.00271

**Published:** 2019-06-19

**Authors:** Rainer Viktor Haberberger, Christine Barry, Nicholas Dominguez, Dusan Matusica

**Affiliations:** ^1^Pain and Pulmonary Neurobiology Laboratory, Centre for Neuroscience, Anatomy and Histology, Flinders University, Adelaide, SA, Australia; ^2^Órama Institute, Flinders University, Adelaide, SA, Australia

**Keywords:** DRG, satellite cells, immunohistochemistry, nociceptor, MRI

## Abstract

Sensory neurons with cell bodies situated in dorsal root ganglia convey information from external or internal sites of the body such as actual or potential harm, temperature or muscle length to the central nervous system. In recent years, large investigative efforts have worked toward an understanding of different types of DRG neurons at transcriptional, translational, and functional levels. These studies most commonly rely on data obtained from laboratory animals. Human DRG, however, have received far less investigative focus over the last 30 years. Nevertheless, knowledge about human sensory neurons is critical for a translational research approach and future therapeutic development. This review aims to summarize both historical and emerging information about the size and location of human DRG, and highlight advances in the understanding of the neurochemical characteristics of human DRG neurons, in particular nociceptive neurons.

## Introduction

Sensory neurons relay information about a variety of intrinsic and environmental cues such as temperature, touch, muscle length, organ volume or actual or potential harm to the body. They also contribute to regulation of blood supply and change neuronal sensitivity and other functions by ortho- and antidromic release of molecules. The cell bodies of sensory neurons are located primarily in dorsal root ganglia (DRG) or trigeminal ganglia (TG; see reviews Belmonte and Viana, 2008; Pope et al., 2013; Krames, 2015; Nascimento et al., 2018). The last three decades have seen significant advances in understanding the electrochemical, cellular and molecular characteristics of sensory neurons found in DRG, primarily stemming from animal studies. These studies have focused heavily on understanding mechanisms underlying the development and pathophysiology of chronic and/or neuropathic pain. Far less, however, is known about the cellular and molecular characteristics of human DRG. The emergence of recent comparative genetic and proteomic studies between animal and human models has highlighted critical differences and similarities in molecular and cellular characteristics of DRG. These may have profound implications for translating data from rodent models to human pathologies, and subsequent therapeutic developments. In view of the large-scale failure of clinical trials based on animal models, the success of new drugs to treat pain in the clinic will likely require studies of human cells and tissues. The emergence of researchers with the capacity to acquire and study native human sensory neurons in the DRG through organ-donor networks, in conjunction with data gained from clinical trials of DRG stimulation for treatment of chronic neuropathic pain, will be critical to validate important pain mechanisms discovered in animal models. There are numerous comprehensive reviews summarizing advances in the understanding of rodent DRG, however, to our knowledge, no reviews focused on collating information on human DRG have been published to date. This review aims to encapsulate existing information about human DRG neurons in relation to their size, location, blood supply, and neurochemical content under non-pathological conditions.

**FIGURE 1:**
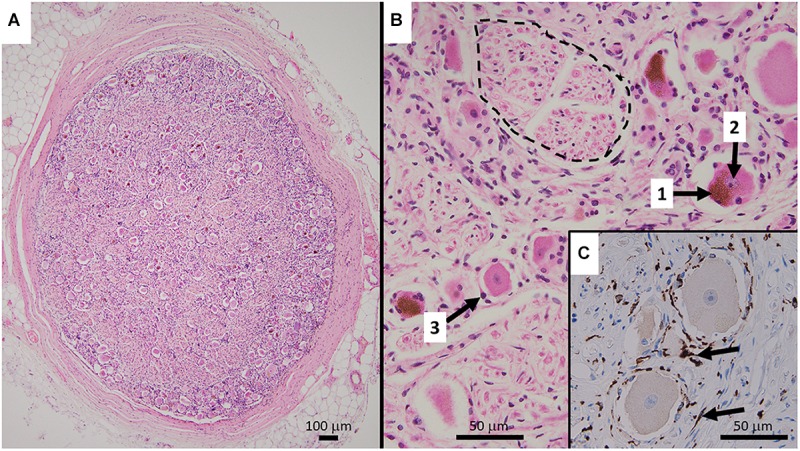
**(A)** Representative HE stained micrograph section of a thoracic human DRG (medical dissection course, ethics approval obtained from The Southern Adelaide Clinical Human Research Ethics Committee, OFR no.: 55.17) with a thick protective layer of connective tissue demonstrating the predominant localization of cell bodies in the periphery of the ovoid DRG cross-section. **(B)** Cell bodies of sensory neurons containing lipofuszin (1) and a nucleus with a prominent nucleolus (2), surrounded by satellite cells (3). Bundles of nerve fibers (dashed line) are predominantly present in the center of the ganglion. The HE staining method results in shrinkage of the cell bodies which disconnects them from the layer of satellite cells. **(C)** Immunohistochemistry micrograph for CD163 with counterstaining for hematoxylin shows the presence and distribution of macrophages (arrows) in DRG.

**FIGURE 2:**
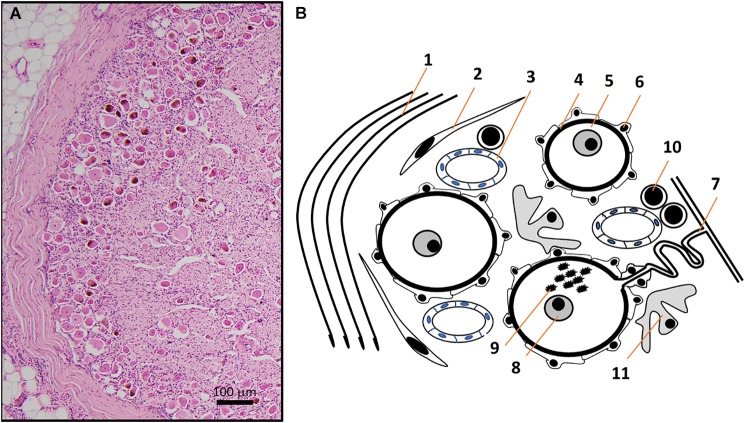
**(A)** HE micrograph section of a thoracic human DRG in higher magnification shows a high number of neuronal somata of different sizes in the periphery of DRG, next to the thick connective tissue covering. **(B)** Schematic representation of the HE micrograph figure highlighting the variety of different structures and cell types in human DRG. Connective tissue layers (Reina et al., 1996) (1), fibroblasts (2), capillaries (Kutcher et al., 2004) (3), basement membrane (Johnson, 1983) (4) between nerve cells (5) and satellite cells (Hanani, 2005) (6). The pseudo-unipolar process (Rudomin, 2002) (7) originates from sensory neurons with prominent nuclei containing a singular nucleolus (Berciano et al., 2007) (8) and sometimes lipofuszin (Moreno-Garcia et al., 2018) (9). Non-neuronal cells in DRG include T- and B-lymphocytes (10) and macrophages (11) (Graus et al., 1990a).

Dorsal root ganglia do not only contain the cell bodies of primary sensory neurons but also a variety of other cell types such as a specific form of glia, called satellite cells, that form a layer (envelope) around neuronal cell bodies (Pannese, 1981; Hanani, 2005, 2010a,b; Takeda et al., 2009). Neurons and satellite cells form a functionally close relationship (Figure 1). Studies on cat DRG demonstrate the presence of microvilli as extensions of the neuronal cell surface, in close contact with surrounding satellite cells (Pannese, 1981). Satellite cells express a characteristic pattern of surface receptors (Hanani, 2005), transporters, and enzymes. Glutamine synthetase and proteins of the S100 family can be used to neurochemically identify these cells (Hanani, 2005). Satellite cells are able to modify the microenvironment of neurons by uptake and release of molecules, but interestingly seem not to have a barrier function (Hanani, 2005). In addition to neurons and satellite cells, DRG contain small blood vessels, thus endothelial and smooth muscle cells, delivering blood to satisfy the extensive energy and therefore oxygen demand of sensory neurons. With neuronal processes as long as a meter, ongoing synthesis and transport of proteins over hundreds of millimeters is critical for normal neuronal function. The blood vessels build an extensive network of arterioles and capillaries within DRG (Kutcher et al., 2004; Kubicek et al., 2010). The interface between accumulations of sensory neurons and blood vessels in DRG is unique. Capillaries in DRG are fenestrated and in the absence of a blood–brain barrier, many blood borne molecules can directly enter the DRG and interact with neuronal and non-neuronal cells (Arvidson, 1979; Kiernan, 1996). Non-neuronal target cells include a group of immune cells contained within DRG that consist mainly of macrophages and T-lymphocytes and a lower number of B-lymphocytes (Schmid et al., 2013; Lakritz et al., 2015; Makker et al., 2017) (Figure 2).

Compared to rodents, human DRG are larger and contain more cells with different proportions of sensory neuron subtypes and substantially more connective tissue between neurons. Recent studies have showed that compared to classical laboratory animals, human sensory neurons contain similar sets of receptor and channel proteins but their expression levels and function of key components relevant to mechanisms underlying chronic pain, such as sodium channels, can differ (Han et al., 2015; Castro et al., 2017; Zhang et al., 2017, 2019). Given that sodium channel blockers are currently in clinical trials for analgesic drug development (Levinson et al., 2012; Thomas and Atkinson, 2018), this further demonstrates the importance of comparative human data. Additional differences are demonstrated by functional studies examining non-neuronal cells in the DRG. Recent animal studies suggest an important function for satellite glia and immune cells in the development of pain. However, only one publication to date addresses the presence of immune cells such as macrophages and lymphocytes in human DRG under non-pathological conditions (Graus et al., 1990a). Shifting focus to the functional characteristics of mast cells in the DRG, there are no human studies. Considering that a small number of recent comparative studies have demonstrated critical rodent and human inter-species differences between the cellular machinery associated with the development of pathological pain conditions, it is fundamentally important to investigate the cellular and molecular components of human DRG to advance our understanding of these diseases. As a first step toward the objective of investigating human DRG, we believe it is pivotal to summarize current existing knowledge in this field. Here, we focus on describing the location and structure of human DRG, and neurochemical characteristics of satellite glia cells (SGCs) and DRG neurons with emphasis on the nociceptor-related neurochemistry.

## Human Dorsal Root Ganglia: Macro-Anatomy

Information about the location and size of human DRG is mainly based on investigations using cadaveric material or studies using magnetic resonance imaging (MRI).

Humans possess 31 pairs of spinal nerves containing, inter alia, sensory nerve fibers with cell bodies in DRG. The number of DRG often equals the number of spinal nerves. Nevertheless, the first cervical (C1) DRG has been shown to be smaller compared to DRG at other vertebral levels, in addition to being present in only about a quarter of investigated bodies (28.5%) (Tubbs et al., 2007). The size of human DRG depends on the vertebral level. Even though there might be size differences between ganglia in individuals, on average, no differences exist between ganglia on the left and right side of the body and no age-dependent differences have been reported (Hasegawa et al., 1996; West et al., 2012; Reinhold et al., 2015; Godel et al., 2017). In the cervical region, DRG size increases from the very small and sometimes absent DRG at the level C1 to larger DRG at the C8 level. The C8 DRG also has a larger volume (177 ± 27.5 mm^3^) than the adjacent T1 DRG (144 ± 30.8 mm^3^) (West et al., 2012). No studies to date have measured human thoracic DRG at lower levels (T3–T12), whereas several studies provide data for DRG at lumbar and sacral levels. Table 1 summarizes studies that have identified various human DRG dimensions: width; width and length; or volume. The size of lumbar DRG increases from vertebral levels L1 to L5, from approximately 3–5 mm × 4–5 mm at L1 to 5–6 mm × 9–11 mm at L5. Conversely, the size of sacral DRG decreases from S1 to S4 with a very large ganglion at level S1 (6–7 mm × 11–12 mm) (Hasegawa et al., 1996; Ebraheim and Lu, 1998) (Table 1). The correlation between DRG size and the number of neurons contained within has also been demonstrated (West et al., 2012).

**Table 1:** Summary table for sizes of human DRGs.

Dorsal root ganglia (mm^3^)			Cervical		Thoracic	Lumbar			Sacral		
References	Method	Participants (*n*)	C7	C8	T1	L3	L4	L5	S1	S2	
West et al., 2012	MRI	19 (14 m and 5 f)	182 ± 16.2	177 ± 27.5	144 ± 30.8						
Godel et al., 2016	MRI	46 (24 m and 22 f)									
Godel et al., 2017	MRI	13 (13 m)				149.26 ± 45.41	182.58 ± 65.78	239.47 ± 74.96	269.90 ± 70.58	163.42 ± 57.49	
Godel et al., 2018	MRI	26 (13 m and 13 f)				124.1 ± 51.7	158.0 ± 59.7	225.2 ± 61.6	230.3 ± 72.8	134.7 ± 57.5	

**Dorsal root ganglia, length/width (mm)**			**Lumbar**					**Sacral**			
		
**References**	**Method**	**Participants (*n*)**	**L1**	**L2**	**L3**	**L4**	**L5**	**S1**	**S2**	**S3**	**S4**

Hasegawa et al., 1996	MRI	20 (20 m)	4.3 ± 0.8	5.7 ± 1.2	7.1 ± 1.3	8.1 ± 1.1	9.4 ± 1.4	11.2 ± 1.7			
			3.7 ± 0.7	4.6 ± 0.7	5.7 ± 0.7	6.2 ± 0.7	5.9 ± 0.7	6.2 ± 0.4			
Shen et al., 2006	MRI	115 (54 m and 61 f)	4.35 ± 0.89	5.85 ± 1.11	7.20 ± 1.36	8.64 ± 1.49	11.58 ± 2.25				
			3.38 ± 0.77	4.51 ± 0.88	5.37 ± 0.96	5.83 ± 0.94	6.40 ± 0.91				
Ebraheim and Lu, 1998	Dissection	20 (9 m and 11 f)						12.8 ± 1.8	10.1 ± 1.5	8.0 ± 1.4	5.1 ± 1.6
								6.3 ± 1.3	5.1 ± 0.9	4.1 ± 0.8	2.9 ± 0.9
Silav et al., 2016	Dissection	15 (gender unspecified)	5.39	5.83	7.24	7.97	10.83				
			4.36	4.56	4.99	5.22	5.82				
Reinhold et al., 2015	MRI T1.5/T3	20 (10 m and 10 f)	7	7.3	7.9	7.9	8	8.9			
			4.6	4.9	5.2	4.8	4.8	5.4			
Reinhold et al., 2015	MRI T1.5/T3	20 (10 m and 10 f)	6.6	7.4	8	7.8	7.9	8.1			
			4.5	5.1	5.4	5.1	5	5.3			
Silverstein et al., 2015	MRI and dissect.	16 (10 m and 6 f)	No data	No data	No data	No data	No data				
			3.2 ± 0.7	4.3 ± 0.4	4.9 ± 0.3	6.1 ± 0.4	6.5 ± 0.5				

**Dorsal root ganglia height (mm)**			**Lumbar**							
**References**	**Method**	**Participants (*n*)**	**L1**	**L2**	**L3**	**L4**	**L5**		

Hasegawa et al., 1996	MRI	20 (20 m)	4.3 ± 0.9	5.6 ± 1.2	7.3 ± 1.4	8.2 ± 0.9					

**Dorsal root ganglia diameter (mm)**			**Lumbar**				**Cervical**			
**References**	**Method**	**Participants (*n*)**	**L4 left**	**L4 right**	**L5 left**	**L5 right**	**C2**				

Zhao et al., 2018	3D CT	23 (15 m and 8 f)	5.8 ± 0.3	5.5 ± 0.4	5.7 ± 0.4	6.1 ± 0.5			
Koeppen et al., 2017	Dissection	12					4.6-4.97

**Dorsal root ganglia area (mm^2^)**		**Lumbar**	**Sacral**					

**References**	**Method**	**Participants (*n*)**	**L5**	**S1**					

Beom et al., 2018	MRI	10 (8 m and 2 f)	66.6 ± 13.7	79.5 ± 14.3					


*m, male; f, female.*

Considering that human DRG are susceptible to damage by compression, e.g., by disc herniation (Weinstein, 1986), the location of DRG in relation to adjacent structures is important. Compared to other DRG, the second cervical (C2) DRG has an unusual relationship to unique adjacent structures such as atlas and axis or the non-bony posterior border created by the ligamentum flavum (Lu and Ebraheim, 1998; Bilge, 2004). Substantial mobility at this intervertebral level might contribute to vulnerability of the ganglion. Besides mobility, the location of blood vessels can also influence DRG function. Alleyne et al. (1998) described the close location to and compression of the C5 cervical ganglion and ventral roots via the vertebral artery which normally lie anterior or lateral to the cervical DRG (Turnbull et al., 1966).

Dorsal root ganglia are normally localized within, or close to, intervertebral foramina, the openings between the pedicles of vertebrae that allow peripheral structures to connect with the vertebral canal. Within the intervertebral foramen, DRG are normally localized superolaterally, but at lower vertebral levels they tend to be positioned more centrally within the foramen. Kikuchi et al. (1994) classified DRG location as intraspinal (in the vertebral canal, proximal to the vertebral foramina), intraforaminal (in the intervertebral foramina) or extraforaminal (distal to the intervertebral foramina). A study investigating C6 and C7 DRG showed that about half were situated extraforaminally (Yabuki and Kikuchi, 1996). Lumbar DRG are predominantly intraforaminal. Sacral DRG are localized more centrally, with DRG at levels S1 and S2 localized intraforaminal and S3 and S4 in the vertebral canal (intraspinal) (Sato and Kikuchi, 1993; Kikuchi et al., 1994; Hasegawa et al., 1996; Ebraheim and Lu, 1998; Moon et al., 2010). No differences have been observed in the location of DRG with respect to gender, age, height, and weight (Moon et al., 2010).

## Human Dorsal Root Ganglia: Micro-Anatomy

Interestingly in humans, individual DRG can occasionally consist of one, two or three smaller and distinctly sheathed ganglia (Kikuchi et al., 1994; Shen et al., 2006). DRG containing two or even three ganglia are predominantly present at vertebral levels L3 and L4 (Shen et al., 2006). Whether this is of functional significance remains unknown.

Human DRG normally consist of a peripheral region that contains the somata of primary sensory neurons and a central region that predominantly contains bundles of nerve fibers (Jimenez-Andrade et al., 2008; Godel et al., 2016) (Figure 1). They are encased by meninges including a thick layer of dura mater, comprised of collagenous connective tissue (Reina et al., 1996). Based on light microscopy and routine hematoxylin and eosin (HE) staining, neuronal cell bodies can be subdivided into small phase-dark neurons and large phase-light neurons, with distinct types of neurons already present at gestational week 6 (Marti et al., 1987). Fluorescence and light microscopy show human DRG neurons often contain highly autofluorescent accumulations of material (fluorescence microscopy) or accumulations of brownish material. This is the pigment lipofuscin which consists of a mixture of lipids, misfolded proteins and sugar molecules (Moreno-Garcia et al., 2018) (Figure 1,2). Human DRG neurons show age-dependent inclusions of lipofuscin, which are accompanied by accumulations of melanin (Scharf and Blumenthal, 1967). The nuclei of human DRG neurons normally contain one nucleolus that is relative in size to the cell body, and possess Cajal bodies, suborganelles that are in involved in RNA processing (Berciano et al., 2007) (Figure 1, 2).

It is difficult to determine the exact number of primary sensory neurons within DRG as the ganglia are not round but rather ovoid and elongated. Furthermore, DRG contain not only neurons but also non-neuronal cells, connective tissue, blood vessels and bundles of nerve fibers. Stereological quantification was used to determine the number of DRG neurons that project with the brachial plexus to peripheral targets. The study determined that about 60,000 neurons are present in DRG at the level of C5 and about 100,000 at the C7 level (West et al., 2012).

Developmentally, all DRG neurons are initially similar in size, and differences in cell size start to appear from week 6 of gestation on Marti et al. (1987). The size of human DRG neurons identified in cryostat sections ranges from approximately 20 to 100 μm in diameter. Human neurons are larger compared to rodent DRG but the distribution of small and large neurons is similar (Josephson et al., 2001; Zhang et al., 2017). Small to medium sized human neurons are considered those with somata of <60 μm (Davidson et al., 2014; Han et al., 2015; Chang et al., 2018). In contrast to human DRG neurons *in situ*, neurons of small to medium sizes dominate in neuronal cultures of dissociated ganglia [36 ± 2.6 μm in diameter (Anand et al., 2016)] as the conditions of isolation probably destroy larger neurons, similar to laboratory animals. In cultured, dissociated human DRG neurons ranged from 28 to 56 μm in diameter (Davidson et al., 2014).

Neurons in DRG possess a T-shaped pseudo-unipolar process that originates from the cell body via an initial segment (Figure 2). Animal studies have shown that it extends with a shorter “axonal” central process connected to and arborizing within the spinal cord dorsal horn (see review, Rudomin, 2002) and a peripheral “axonal” process innervating target tissues. Data in relation to the central sensory fiber arborization in human spinal cord do not exist. Peripheral processes have been described in different human tissues. Some processes are only one micrometer in diameter but travel large distances. The peripheral process from a lumbar DRG neuron that innervates for example the big toe, can exceed 1 m = 1,000,000 μm. This presents a massive extension of the cell, restricting only 1–2% of the cytosol to the cell body with the majority of cytosol and cytoskeletal elements present in pseudo-unipolar processes (see review, Devor, 1999).

The initial segment of the pseudo-unipolar process of human DRG neurons is elongated and it forms a glomerulus-like structure (Figure 2). With age, the structure is increasingly surrounded by glial fibrillary acidic protein (GFAP)-positive SGCs (Murayama et al., 1991) but neurons and satellite glia are still separated by a basement membrane approximately 100 nm thick (Johnson, 1983).

## Human Dorsal Root Ganglia: Blood Supply

Dorsal root ganglia are situated outside of the blood brain barrier and animal studies clearly show the presence of fenestrated capillaries (Figure 2). Hence, molecules circulating in the vascular system can directly access to the DRG. This vascular organization provides the human DRG with a robust blood supply, serving neurons that have long processes with the required high-energy demand critical for maintaining the production and transport of receptors, ion channels, cytoskeletal and transport proteins. Two interconnected arterial plexuses, situated superficially and deep, supply human DRG. These plexuses originate from arteries that derive from the radiculomedullary branches of segmental arteries (Yoshizawa et al., 1991; Gilchrist et al., 2002; Parke and Whalen, 2002). Peri-ganglionic venous plexuses drain predominantly from the dorsal side of DRG into intervertebral veins (Takano et al., 1998; Parke and Whalen, 2002). Recently, Godel et al. (2016, 2017) used dynamic-contrast-enhanced MRI perfusion to investigate the dynamics of blood supply of human DRG. The blood supply *in vivo*, expressed as perfusion of DRG determined by blood-tissue permeability and interstitial leakage fraction, was higher in DRG compared to spinal nerves. Interestingly the perfusion of DRG was significantly higher in women compared to men (Godel et al., 2016, 2017).

## Satellite Glial Cells (SGCs)

This specialized group of DRG and TG-specific glia cells (Hanani, 2005, 2010a,b) not only surround the initial segment, but build an envelope around the somata of nerve cells (Figure 1, 2) and are able to modulate neuronal function (Pannese, 1981; Hanani, 2005, 2010a,b). Animal studies to date have revealed a substantial amount of receptors, transporters and ion channels are expressed in SGC (see review, Hanani, 2005). Unfortunately, only a small number of studies have investigated the neurochemical characteristic of human SGC (Table 2). Those studies show that, similar to those of laboratory animals, human glia cells exhibit characteristic immunoreactivities for S100 beta protein and glutamine synthetase (Pan et al., 2012; Koeppen et al., 2016). Koeppen et al. (2016) performed a carefully controlled immunohistochemical analysis of characteristic molecules in human SGCs, and showed the presence of the metabotropic glutamate receptor 2/3, (mGlu2/3), the ATP-sensitive inward rectifying potassium channel 1.4 (Kir 1.4) and the excitatory amino acid transporter 1 (EAAT1) in human SGCs (Koeppen et al., 2016) (Table 2). Since the primary transmitter of nociceptive DRG neurons is glutamate, the presence of mGlu2/3 and EAAT1 suggest active involvement of SGC in the glutamate turnover of human DRG neurons. Animal studies showing inter-SGC connections and the presence of connexin 43, a major component of gap-junctions (Koeppen et al., 2016), suggest SGC involvement in cells-to-cell communication. Recently, Li et al. (2018) demonstrated immunoreactivity for Na_V_1.7 in GFAP-positive putative SGCs within DRG removed from patients with cancer-related neuropathic pain. It remains unknown if this “nociceptive” ion channel is present in SGC under normal conditions. Nevertheless, immunoreactivity for glial-derived neurotrophic factor (GDNF), another molecule relevant for both DRG development and pain signaling, is present in SGC (Bar et al., 1998).

**Table 2:** Molecules described in human satellite glial cells using immunohistochemistry.

Antigen	Host, supplier	References	Specificity control	Number	Source
S100	Mouse; Santa Cruz	Koeppen et al., 2016	Preabsorption	12 (gender unspecified)	Autopsy
Glutamate synthetase	Mouse; Santa Cruz	Koeppen et al., 2016	Preabsorption	12 (gender unspecified)	Autopsy
Metabotropic glutamate receptor 2/3 (mGluR2/3)	Rabbit; Novus	Koeppen et al., 2016	Preabsorption	12 (gender unspecified)	Autopsy
ATP-sensitive potassium channel 1.4 (Kir 1.4)	Rabbit; Alomone	Koeppen et al., 2016	Preabsorption	12 (gender unspecified)	Autopsy
Excitatory amino acid transporter 1 (EAAT1)	Goat; Santa Cruz	Koeppen et al., 2016	Validated via MIDAS	12 (gender unspecified)	Autopsy
Connexin 43	Mouse; Abcam	Koeppen et al., 2016	Validated via MIDAS	12 (gender unspecified)	Autopsy
Voltage-gated sodium channel 1.7 (Na_V_1.7)	Rabbit; Alomone	Li et al., 2018	Preabsorption	6 DRG, 3 donors (2 m and 1 f)	Spinal surgery
Glial-derived neurotrophic factor (GDNF)	Not specified; Santa Cruz	Bar et al., 1998	Preabsorption	5 DRG, 5 donors (gender unspecified)	Autopsy


*Specificity control: Accepted as evidence supporting the specificity of the primary antiserum/antibody were preabsorption with the corresponding antigen and Western blot showing the complete blot with a single band at the correct molecular weight. Evidence considered not specific for immunoreactivity in human DRG included absence of immunoreactivity in antigen-deficient (knock-out) cells or positive staining in target tissues in animals.*

Evidence indicates satellite cells recognize foreign molecules and participate in immune-mediated processes. Earlier studies showed that SGC in human DRG express class I and II Major Histocompatibility Complex (Graus et al., 1990a,b). More recently, studies in human SGC obtained from TG have demonstrated a class of pathogen- and damage-associated pattern recognition receptors, the Toll-like receptors (Mitterreiter et al., 2017). Studies characterizing subpopulations of DRG neurons through the differential expression of surface carbohydrate antigens discovered that SGC express gangliosides such as the GD1b ganglioside and fucosyl GM1 (Kusunoki et al., 1989, 1991, 1997). Similarly, interest in expression of molecules in DRG neurons demonstrated the expression of a variety of molecules in SGC, such as the EGF receptor and the Bcl-2 homologous antagonist Bak (Huerta et al., 1996; Krajewski et al., 1996). SGCs also express the olfactory receptor 6B2 protein (Flegel et al., 2015). In summary, human SGCs share many features with SGCs of small laboratory animals, such as the expression of glia- and neuron-related molecules. But compared to results of animal experiments human data is limited and taking into account the established close relationship between sensory neurons and DRG-specific glia, a lot of work needs to be done to further characterize this important cell type in human DRGs.

## Neurochemical Characteristics of Human DRG Neurons

Dorsal root ganglia contain a complex array of sensory neuron cell bodies that have different functions and innervate different targets. Many proteins such as channel proteins are highly related to neuron function. The method capable of identifying protein localization in complex tissues with high resolution is immunohistochemistry. This section aims to provide data for normal, uninjured human DRG immunohistochemistry, and does not focus on molecules related to any form of pathology. Only data obtained from studies of normal human DRG and from studies using normal human DRG as control tissues are discussed in this segment.

As noted previously, human DRG neurons measure between about 20 and 100 μm in diameter (Holford et al., 1994; Coward et al., 2000; Anand et al., 2006; Li et al., 2018) and occupy areas between 1,500 and 5,000 μm^2^ (Koeppen et al., 2011). There are no reliable and distinct morphological characteristics that allow the functional subdivision of sensory neuron subpopulations based on neuronal soma size or shape. However, neurochemical characteristics represent the most commonly used method to describe aspects of neuronal function and classification in the DRG. The emergence of genomic and transcriptomic studies has recently opened a window for researchers to re-evaluate the classification of DRG neuron subtypes based on mRNA expression patterns in individual rodent neurons (Usoskin et al., 2015). However, further validation of these groundbreaking studies will be required before a more advanced classification systems is established and widely accepted. This section will focus on neurochemical characteristics of human DRG neurons, starting with the description of common neuronal markers with an overarching focus on neurochemical characteristics associated with nociceptive function. Given the relative wealth of data included in this section, Supplementary Table 1 provides an appended overview of critical variables, including the number of participants or DRG donors, and more importantly the presence or absence of controls used for the specificity of the primary antisera/antibodies (Saper, 2009).

### Common Neuronal Markers

#### Neurofilaments (NFs)

Neurofilaments (NFs) are cytoskeletal intermediate filaments of varying molecular weights ranging from ∼56 to 200 kDa. They provide structural support and regulate axonal diameter, and are present in all neurons, including the DRG. In rodents, NF200, a neurofilament of 200 kDa, labels a population of larger, myelinated A-fiber neurons. Human studies have shown the presence of neurofilaments in DRG neurons early in development. Immunoreactivity for a neurofilament 150 kDa was present in fetal DRG neurons at week 6 (Marti et al., 1987). Neurofilament 200 immunoreactivity was detected in few cells in week 10 but in all neurons at weeks 17–18 of gestation (Suburo et al., 1992). This is also true for adult human DRG where, in contrast to rodents, NF200 immunoreactivity is not restricted to larger neurons but present in virtually all DRG neurons (Suburo et al., 1992; Naves et al., 1996; Rostock et al., 2017; Chang et al., 2018). Similarly, most or all adult human DRG neurons show immunoreactivity for other cytoskeletal molecules such as α- and β-tubulin, microtubule-associated proteins 1 and 5, and neurofilaments 68 and 160 (Naves et al., 1996).

#### PGP9.5 (UCHL1)

PGP9.5 (UCHL1), a ubiquitin C-terminal hydrolase is present in all neurons and is a pan-neuronal marker. This protein has emerged as one of the key neuronal markers in rodents and humans, and is used to differentiate between neuronal and non-neuronal structures in DRG (Coward et al., 2000; Anand et al., 2018; Desormeaux et al., 2018).

#### Tuj1 (Beta3 Tubulin, TUBB3)

Tuj1 (beta3 tubulin, TUBB3) is part of microtubule element within the tubulin family found predominantly in neurons and testes in healthy tissues. Detection of this protein is widely used as a pan-neuronal marker in immunohistochemical studies, as it readily differentiates neuronal from glial cells, which do not express beta3 tubulin. It is primarily detected in somata and processes of human DRG neurons *in situ* and under culture conditions (Naves et al., 1996; Robinson et al., 2007; Enright et al., 2016; Rostock et al., 2017; Anand et al., 2018).

#### Peripherin (PRPH)

Peripherin (PRPH) is an intermediate filament protein that is expressed in neurons of the peripheral nervous system such as small DRG neurons and in central neurons that innervate peripheral targets such as motor neurons (Bae et al., 2015).

#### Brn3a (Pou4f1)

Brn3a (Pou4f1), a sensory neuron marker, is a POU homeodomain transcription factor that regulates gene expression and differentiation of sensory neurons. In animal studies it has been shown to be expressed from nearly all sensory DRG neurons (Badea et al., 2012). It has been found present in all human DRG neurons of adults (Rostock et al., 2017) and gestational weeks 9–11 (Schonemann et al., 2012), and has been found colocalized with NF200 (Rostock et al., 2017).

#### NeuN (RBFOX3)

NeuN (RBFOX3) is a RNA binding protein found predominantly in the neuronal nuclei, and another common neuronal biomarker found in the vast majority of postmitotic (mature) neurons (Duan et al., 2016). In the limited studies performed to date, NeuN is expressed in all adult human DRG neurons but was not detected at gestational week 10 (Schonemann et al., 2012).

### Molecules Characteristic of Nociceptors

#### Ion Channels

Ion channels are essential for the regulation of neuronal excitability leading to the generation and conduction of action potentials and are therefore critical to sensory neuron function. Important channel proteins for the generation of inward membrane currents in nociceptors belong to the groups of voltage-gated sodium (Na_V_) and calcium (Ca_V_) channels as well as transient receptor potential (TRP) channels (Waxman and Zamponi, 2014). Given the sensory nature of the DRG, and their role in the development of chronic pain conditions, it’s not surprising that in relation to the detection of channel proteins in individual neurons, human DRG studies have predominantly focused on channels related to nociception.

##### Voltage-activated sodium channels

Voltage-activated sodium channels are key components of action potential generation in DRG neurons. Out of the nine Na_V_ subtypes, the Na_V_ 1.7, 1.8, and 1.9 are of particular interest as they have established roles as key components of pain signaling events (Waxman and Zamponi, 2014; Namer et al., 2015; Dib-Hajj et al., 2017). Studies using multiple labeling immunohistochemistry (Coward et al., 2000, 2001; Li et al., 2018) have demonstrated that immunoreactivity for the voltage-gated sodium channel Na_V_1.7 is present in about half of all DRG neurons. In contrast to animal studies, in humans the Na_V_1.7 immunoreactivity is not restricted to small neurons but present in neurons of all sizes (Li et al., 2018). Immunoreactivities for Na_V_1.7 (PN1), Na_V_1.8 (SNS/PN3) and Na_V_ 1.9 (NaN/SNS2) have been detected in all small (<30 μm), medium-sized (<50–55 μm) and large (>55 μm) neurons (Coward et al., 2000, 2001), which also suggests the presence of voltage-gated sodium channels Na_V_ 1.7, 1.8, and 1.9 in neurons of all sizes. Nevertheless, the strongest immunoreactivity was detected in small neurons (Coward et al., 2000, 2001).

A recent study investigating the presence of Na_V_ 1.6, 1.7, 1.8, and 1.9 in human DRG using *in situ* hybridization (Rostock et al., 2017), has shown that all Na_V_ channels were present in human DRG and expressed in presumably nerve growth factor-dependent, TrkA expressing neurons. The Na_V_ 1.9 showed the lowest level of colocalization with TrkA (present in about 25% of neurons), the Na_V_1.8 the highest (about 70% of neurons). Interestingly, the study directly compared proportions of positive cells in human and mouse DRG and showed significant differences between mouse and humans for Na_V_1.8 and Na_V_1.9 (Rostock et al., 2017).

These findings are supported by RT-PCR and RNAseq studies that demonstrated the expression of mRNA for Na_V_ subunits in human DRG explants (Dib-Hajj et al., 1999; Jeong et al., 2000; Chang et al., 2018; Ray et al., 2018). Interestingly, qRT-PCR showed that the expression levels and proportions of Na_V_ channel subtypes were different between humans and mice (Chang et al., 2018). The expression of the Na_V_ 1.8 channel subtype gene SCN10A, was much lower in human DRG, when compared to gene expression levels reported in mouse studies. In contrast, the expression of the Na_V_1.7 channel subtype gene SCN9A, was much higher in human than mouse samples (Chang et al., 2018). These findings are supported by an RNAseq study (Ray et al., 2018) that found channel subunit SCN9A expression levels are more abundant in human DRG compared to mouse data. The sequencing data support qRT-PCR data and show differences in expression levels between mice and human DRG.

In addition to transcriptional and translational data, electrophysiological studies confirm the functional presence of Na_V_1.7 and Na_V_1.8 channels in human DRG neurons. Sensitivity to the puffer fish toxin tetrodotoxin (TTX) selectively differentiates between channel subtypes, where Na_V_1.8 and 1.9 are TTX-resistant, Na_V_1.1, 1.2, 1.3, 1.6, and 1.7 are TTX-sensitive (Waxman and Zamponi, 2014). Human DRG neurons possess TTX-sensitive and TTX-resistant channels, but in contrast to rodents, where TTX-resistant currents are mainly restricted to small diameter neurons, in humans they are present in small and large diameter neurons (Han et al., 2015; Zhang et al., 2017). Nevertheless, the Na_V_1.7 channel is the major TTX-sensitive sodium channel in DRGs in both humans and mice (Alexandrou et al., 2016). However, in humans, Na_V_1.8 channels exhibit different functional properties and the density of TTX-sensitive and -resistant channels is much higher than that detected in animal studies (Han et al., 2015; Zhang et al., 2017).

##### Voltage-gated calcium channels (Ca_V_)

Voltage-gated calcium channels (Ca_V_) are essential components of sensory neuron function (Park and Luo, 2010) as activation of these channels contributes to exocytosis of transmitter-filled vesicles at synaptic endings. The channels can be subdivided based on different criteria such as high-voltage activated and low voltage activated (HVA, LVA), similarity of the α1-subunit (Ca_V_1, Ca_V_2, Ca_V_3) or sensitivity to pharmacological inhibitors (L, N, P/Q, R, T) (Lacinova, 2005; Park and Luo, 2010). To date no studies have reported the cellular location of Ca_V_ proteins in human DRG neurons, however, the expression of Ca_V_-mRNA has been confirmed by RT-PCR as well as single cell PCR. Interestingly, the Ca_V_2.2 channel was expressed in 56.4% of cultured human DRG neurons whereas the Ca_V_2.3 was found to be expressed only in 5% of neurons (Castro et al., 2017).

##### Calcium-activated potassium channels (K_Ca_)

Calcium-activated potassium channels (K_Ca_) are important contributors to the after hyper-polarization of neurons which can be modulated by NMDA-type glutamate receptor activation and nerve ligation, and therefore contribute to nociceptive signaling (Li et al., 2007; Pagadala et al., 2013). Immunoreactivities for voltage-independent human K_Ca_2.1 (SK1) and K_Ca_3.1 (IK1) channels were shown in almost all (between 87% and 95%) sensory DRG neurons independent of size (Boettger et al., 2002).

##### Purinergic receptor (P_2_X)

Purinergic receptor (P_2_X) subunits, P_2_X_2_ and P_2_X_3,_ can build homo- or heterotrimeric ligand-gated ion channels that are activated by ATP. The channels are part of a variety of neuronal signaling pathways including nociception. Studies in rodents have established that P_2_X_2_ and P_2_X_3_ channels are expressed in DRG neurons with the P_2_X_3_ subunit predominantly expressed in non-peptidergic, GDNF-dependent but NGF-independent nociceptors (Mo et al., 2009).

Interestingly, mRNA for the P_2_X_2_ subunit was reported to be absent in human DRG (Serrano et al., 2012) whereas P_2_X_3_ mRNA and protein were clearly detected in human DRG (Yiangou et al., 2000; Pan et al., 2012; Serrano et al., 2012). Using a carefully tested antiserum, Pan et al. (2012) confirmed the presence of P_2_X_3_-immunoreactivity in virtually all DRG neurons, independent of size. Pan et al. (2012) and Yiangou et al. (2000) demonstrated the presence of strong P_2_X_3_-immunoreactivity only in small to medium sized neurons which, similar to rodents, usually do not express the nociceptor subtype-defining NGF receptor TrkA.

The functional validation of P_2_X channels in human DRG neurons was also supported by electrophysiological studies and Ca^2+^-imaging. However, in this instance only a subgroup of small-sized (30–60 μm in diameter) isolated human DRG neurons responded to ATP with action potential discharges, and increase in intracellular Ca^2+^ levels (Davidson et al., 2014; Enright et al., 2016).

##### Transient receptor potential cation channel subfamily V member 1 (TRPV1)

Transient receptor potential cation channel subfamily V member 1 (TRPV1) is a channel protein that is activated by the vanilloid capsaicin, an ingredient of hot chili peppers, by low pH and noxious heat. Endogenous agonists are endocannabinoids such as anandamide and *N*-arachidonoyl-dopamine (Suh and Oh, 2005). TRPV1 is a non-selective cation channel that has been shown to be an important component of nociceptive signaling (Suh and Oh, 2005). Capsaicin induces pain in humans (Simone et al., 1989), but also induces desensitization of TRPV1 channels and modulates nociceptor function. Consequently, topical capsaicin is currently being successfully used in treatment of pain conditions such as postherpetic neuralgia (Anand and Bley, 2011).

As has been done in animal studies, immunohistochemical studies have confirmed the presence of the TRPV1 protein in human DRG neurons. Interestingly, most studies describe the presence of immunoreactivity not only in small-sized neurons but also in medium and some studies in large-sized somata (Lauria et al., 2006; Facer et al., 2007; Li et al., 2015, 2018; Anand et al., 2016; Chang et al., 2018). The average diameter of TRPV1-immunoreactive neurons was reported as 44 ± 7 μm (Chang et al., 2018). TRPV1 expression was also validated in cultured human DRG neurons (Anand et al., 2008; Enright et al., 2016; Valtcheva et al., 2016), with a high proportion of neurons showing TRPV1-immunoreactivity (Anand et al., 2008).

TRPV1 mRNA expression was detected in human DRG explants (Cortright et al., 2001; Flegel et al., 2015; Ray et al., 2018; Sheahan et al., 2018; Snyder et al., 2018) and cultured human DRG (Han et al., 2016). Functional evidence for the presence of TRPV1 channels in human DRG neurons has been provided using capsaicin, a TRPV1 agonist. Capsaicin activation of cultured human DRG neurons induced action potential discharge and increased intracellular Ca^2+^ levels (Baumann et al., 1996; Li et al., 2015; Anand et al., 2016; Han et al., 2016; Valtcheva et al., 2016; Sheahan et al., 2018).

##### Transient receptor potential cation channel ankyrin 1 (TRPA1)

Transient receptor potential cation channel ankyrin 1 (TRPA1) is a channel protein activated by mustard oil and cinnamaldehyde, and plays an important role as an irritant sensor of a vast amount of compounds in nociceptive signaling, with its expression confirmed in animal DRG neurons (Chen and Hackos, 2015). Immunohistochemistry showed the presence of TRPA1 immunoreactivity in 20% of human DRG neurons, predominantly in small-medium sized (<50 μm) neurons with some staining in larger neurons. Most of the TRPA1 positive cells were also immunoreactive for TRPV1 (Anand et al., 2008). Cultured human DRG neurons obtained from patients with avulsion injury, responded to cinnamaldehyde with an increase in intracellular Ca^2+^ levels (Anand et al., 2008). Additionally, the TRPA1 channel has also been detected in human DRG via *in situ* hybridization, which demonstrated that TRPA1 mRNA expressing cells also express TRPV1 mRNA (Rostock et al., 2017).

#### Peptides

Neuropeptides such as CGRP, SP and galanin are neuromodulators that are co-released with transmitters at the central and peripheral terminals of sensory neurons. In addition to being important cellular markers used in identifying subpopulations of sensory neurons, they are also fundamental contributors to nociceptor function.

##### Calcitonin-gene-related-peptide (CGRP)

Calcitonin-gene-related-peptide (CGRP) is a neuropeptide composed of 37 amino acids. Two isoforms of the peptide exist (α-CGRP and β-CGRP) encoded from two separate genes. CGRP interacts with heteromeric receptors consisting of the calcitonin-receptor-like-receptor (CRLR) and receptor activity-modifying proteins (RAMPs). Although the peptide is a strong arterial vasodilator, it also plays a major role in nociception (Marti et al., 1987; Suburo et al., 1992; Nordlind et al., 2000; Shi et al., 2008, 2012; Patil et al., 2010; Yarwood et al., 2017; Li et al., 2018). Furthermore, CGRP is widely used to define a subpopulation of nociceptive DRG neurons.

Immunoreactivity for CGRP is present in human DRG neurons from early fetal life (Marti et al., 1987; Suburo et al., 1992; Nordlind et al., 2000; Shi et al., 2008, 2012; Patil et al., 2010; Yarwood et al., 2017; Li et al., 2018), initially appearing at weeks 14–16, with staining intensity in DRG neurons increasing between 5 months and adulthood (Pan et al., 2012). Subpopulations of CGRP-containing neurons possess immunoreactivity for other peptides such as substance P or signaling molecules such as TRPV1 and phospholipase C beta 3 (PLCβ3) (Shi et al., 2008) or angiotensin II (Patil et al., 2010).

*In situ* hybridization confirmed the presence of CGRP mRNA in 50–70% of DRG neurons (Giaid et al., 1989; Landry et al., 2003), predominantly but not exclusively in small-medium sized neurons. Some studies have identified only about 20% of DRG neurons showing CGRP-immunoreactivity (Giaid et al., 1989), although others placed the reported proportion as high as 60% (Nordlind et al., 2000).

##### Substance P (SP)

Substance P (SP) is a neuropeptide composed of 11 amino acids. It is synthesized by alternative splicing from a larger precursor mRNA, preprotachykinin-A, coded by the TAC1 gene. It selectively binds to the neurokinin 1 receptor present on nociceptive projection neurons in the rat spinal cord dorsal horn and causes enhanced synaptic activity (Gautam et al., 2016). Animal studies demonstrate a clear involvement of SP in nociceptive signaling, however, in humans, the evidence to date is not as convincing (Babenko et al., 1999; Hill, 2000). SP is present in human DRG, with pre-protachykinin mRNA detected in small-sized neurons representing 10% of all DRG, whereas the proportion of human DRG neurons reported to show immunoreactivity for the peptide varies from 5 to 60% (Giaid et al., 1989; Nagao et al., 1994; Nordlind et al., 2000; Landry et al., 2003). Similarly, SP immunoreactivity is present early in development in neuronal cell bodies of fetal DRG but reports vary. Marti et al. (1987) detected immunoreactivity from week 24 onward, whereas Suburo et al. (1992) reported the presence of SP immunoreactivity from week 11 onward (Marti et al., 1987; Suburo et al., 1992). Despite the apparent similarities between humans and rodents, with both having a subpopulation of nociceptive DRG neurons containing SP, blockade of the action of SP via inhibition of neurokinin 1 receptors is effective in relieving pain in mice (Laird et al., 2000; Borbely et al., 2013), but has failed to generate analgesia in human clinical trials (Hill, 2000).

##### Galanin

Galanin is a peptide consisting of 29/30 amino acids (Lang et al., 2015). Galanin modulates the excitability of dorsal horn neurons and the presynaptic release of glutamate from primary afferents (see review, Lang et al., 2015). It is present in DRG of laboratory animals (Ch’ng et al., 1985; Lang et al., 2015). Galanin mRNA has been detected in human DRG via *in situ* hybridization in a small subpopulation (12.5% ± 1.4) of small-sized (<1550 μm^2^) neurons. The majority of galanin mRNA-expressing neurons also contained CGRP-mRNA (Landry et al., 2003).

##### Somatostatin and its receptors

Somatostatin is a neuropeptide of either 14 or 28 amino acids in length, generated from a precursor peptide and is involved in pain processing via interaction with its cognate receptors producing inhibitory, analgesic effects (Mollenholt et al., 1994). More recent studies suggest that somatostatin is also involved in the signaling of itch (Huang et al., 2018).

Dorsal root ganglia neurons with immunoreactivity for somatostatin are present by gestational weeks 9 and 10, and a small population of immunoreactive cells are detectable throughout all fetal stages, with enduring expression within cells present in DRG of 4-month-old infants (Charnay et al., 1987; Marti et al., 1987). Furthermore, somatostatin immunoreactivity is also present in a subpopulation (17%) of adult human DRG neurons (Nagao et al., 1994; Shi et al., 2014) whereas the somatostatin_2A_ receptor is present only in few neurons in human DRG (Shi et al., 2014).

##### Endothelin-1 (ET1)

Endothelin-1 (ET1) is one of three peptide isoforms, 21 amino acids in length, which act as vasoconstrictors but also induce pruritus and pain (Smith et al., 2014). The ET1 peptide is elevated in patients suffering from sickle cell disease, which is associated with episodes of severe pain and animal studies showed that absence of the ET_A_ receptor subtype blocked sickle cells disease-related pain behavior (Lutz et al., 2018).

In human DRG 30% of neurons show ET1 immunoreactivity (Giaid et al., 1989). *In situ* hybridization shows the presence of ET1 in 75% of large and small DRG neurons. The mRNA for ET1 has been reported to be often colocalized with mRNAs for preprotachykinin or CGRP, where all preprotachykinin mRNA expressing DRG neurons contained mRNA for ET1 (Giaid et al., 1989).

##### Angiotensin II and its receptors

The eight amino acids long peptide angiotensin II is part of the renin–angiotensin–aldosterone system (RAAS) that controls water and electrolyte balance and therefore blood pressure. This peptide also contributes to the regulation of nociception. Animal studies show intrathecally applied angiotensin II elicits nociceptive behavioral responses (Cridland and Henry, 1988; Nemoto et al., 2013), suggesting that angiotensin II released by central projections of DRG neurons may contribute to nociception. In human DRG, the presence of angiotensin II has to date been demonstrated by radio-immuno-assay, HPLC and immunolabeling (Patil et al., 2010; Anand et al., 2013). Angiotensin II immunoreactivity was observed in 75% of small and medium-sized human DRG neurons (Anand et al., 2015). Double-labeling of human DRG showed angiotensin II in neurons expressing CGRP, synaptophysin and cathepsin D (Patil et al., 2010).

There is also evidence that human DRG neurons themselves respond to angiotensin II. The angiotensin II type 2 receptor (AT_2_R) has been identified in cultured human DRG and in 60% of small and medium diameter neurons in immunolabeled sections of human DRG (Anand et al., 2013). Multiple labeling studies affirm the expression of angiotensin II, AT_2_R and TRPV1 in the same neurons (Anand et al., 2015), where over 40% of TRPV1-positive neurons expressed AT_2_R (Anand et al., 2013). Direct involvement of angiotensin II in pain signaling pathways, was supported by experiments where angiotensin II treatment of cultured human DRG neurons increased their response to capsaicin, whereas treatment with an AT_2_R antagonist reduced capsaicin responses (Anand et al., 2013). However, more recently the presence of AT_2_R in human and rodent DRG has been disputed, by studies finding no evidence of mRNA for AT_2_R genes in mouse DRG (Shepherd et al., 2018). Instead, these reports that mechanical pain hypersensitivity induced by angiotensin II is mediated by macrophage AT_2_R, leading to production of reactive nitrogen/oxygen species that in turn activate neuronal TRPA1 (Shepherd et al., 2018). Whether this occurs via a direct action on neurons, or via effects on immune cell-neuron interactions remains to be determined. Despite the uncertainty surrounding their mechanism of action, AT_2_R antagonists are being used as effective analgesics in humans and laboratory animals (Rice et al., 2014; Bessaguet et al., 2016; Shepherd et al., 2018).

#### Other Markers of Sensory and Nociceptive Neurons

##### Isolectin B4 (I-B4)

Isolectin B4 (I-B4) is a plant lectin isolated from *Griffonia simplicifolia*, which labels a subpopulation of nociceptive DRG neurons. In mice, these neurons have been shown represent the group of GDNF-dependent, non-peptidergic nociceptors (Bogen et al., 2015). Although Davidson et al. (2014) noted that they could not detect I-B4 staining in cultured human DRG neurons, others have readily demonstrated I-B4 staining in sections of human DRG (Shi et al., 2008, 2014; Pan et al., 2012). However, it is known that control for the specificity of lectin binding in human sections is difficult, and this is further highlighted in these studies reporting varying detection between membrane and cytosolic I-B4 staining identified by different research groups. On the other hand, mRNA for the gene of the binding partner of I-B4, versican (Bogen et al., 2005, 2015), is expressed in human DRG explants (Ray et al., 2018), but whether the versican V2 isoform that binds I-B4 is produced in human DRG remains to be confirmed.

##### Neurotrophins

Neurotrophins are a family of neurotrophic factors that includes nerve growth factor (NGF), brain-derived neurotrophic factor (BDNF), neurotrophin-3 (NT-3), and neurotrophin-4/5 (NT-4/5). Receptors for these factors include the tropomyosin receptor kinases (Trk) A, B, and C, and the low affinity receptor p75 (Lewin and Nykjaer, 2014). NGF and the activation of its cognate receptor TrkA, is a key factor in the development of DRG neurons, but also critical for the induction of hyperalgesia and pain via modulation of signaling events in adult DRG neurons. Neurotrophin receptors are present in DRG from early in development through to adulthood, however, the dynamics of receptor expression patterns from development to adulthood remain to be studied. Immunoreactivity for TrkB and TrkC is present in human fetal DRG at gestational weeks 9–11 (Schonemann et al., 2012), however, the confirmation of immunoreactivity for their respective ligands BDNF and NT-3 is still lacking.

Glial-derived neurotrophic factor is another neurotrophic factor which, after interaction with its receptors RET proto-oncogene tyrosine kinase (RET) and co-receptor GFRalpha1, modulates a subpopulation of nociceptive, I-B4 binding neurons. The effect of GDNF is complex. Similar to NGF, GDNF is a key factor in the development of DRG neurons, in particular nociceptive neurons but it also impacts adult DRG neurons. In neuropathic pain animal models, intrathecal GDNF reversed pain behavior (Boucher et al., 2000), but GDNF injected into rat muscle induced prolonged hyperalgesia (Alvarez et al., 2012).

Nerve growth factor and its receptors have been described in human DRG (Vega et al., 1994; Anand et al., 1997; Widenfalk et al., 1999; Rostock et al., 2017). Immunohistochemistry shows NGF-like immunoreactivity in small cells, whereas TrkA was present in 65% of DRG neurons with the expression of the receptor distributed between both small and medium-sized cells. More recently, the presence of TrkA, B, and C proteins in human DRG has been described, with almost no overlap of immunoreactivities between TrkA and TrkB, or between TrkA and TrkC-positive cells. TrkA immunoreactivity was localized to small cells with cell bodies measuring between 400 and 800 μm^2^, whereas TrkB and TrkC immunoreactivity was detected in cells with areas much larger cell bodies (the majority around 1,400 and 1,600 μm^2^). As with animal studies, a substantial proportion (about 50%) of human TrkA positive cells express TRPV1 (Rostock et al., 2017). In addition to NGF and its receptors, immunoreactivity for both GDNF protein and one of its receptors, RET, has been detected at 25% and 37% respectively in cell bodies of all sizes (Bar et al., 1998).

The validation of protein expression is further supported by the detection of mRNA in numerous studies reporting high levels of mRNAs for neurotrophins and their receptors, with NGF, BDNF, NT-3 and GDNF, p75 and TrkA, TrkB and TrkC having been validated to date (Yamamoto et al., 1996; Widenfalk et al., 1999; Josephson et al., 2001). Detection of mRNA via *in situ* hybridization for the GDNF receptors RET and GFRalpha 1–3 in human DRG neurons has been also reported in multiple studies (Josephson et al., 2001; Rostock et al., 2017) with RET present in about 70% of investigated neurons (Josephson et al., 2001).

The size of DRG neurons immunoreactive for NGF and GDNF receptors, compared to BDNF receptors was not different (Josephson et al., 2001). The response to NGF and GDNF determines the fate of nociceptors, at least in laboratory animals, where neurons develop into NGF- or GDNF-dependent subtypes with different neuropeptide expression patterns (Molliver et al., 1997). Therefore, it is of fundamental interest to determine factors that drive neurotrophin receptor mRNA expression in development. To date, receptor mRNAs for p75, TrkA-C, as well as the GDNF receptors RET and GFRalpha3 have been validated in fetal DRG at gestational age weeks 9–11 with additional presence of TrkA and TrkB (Widenfalk et al., 1999; Josephson et al., 2001). In contrast to adult DRG, BDNF and neurotrophin-3 (NT-3) mRNAs were detected in fetal DRG.

The roles of NGF and GDNF in rodent DRG neurons have become more clearly defined, especially in driving cell differentiation, survival and target innervation. However, in humans, NGF and GDNF appear to also modulate the size of cultured human DRG neurons obtained from patients with brachial plexus avulsion. Presence of these growth factors increased the mean diameter of cultured human cervical DRG neurons significantly from 42 ± 4 μm to 62 ± 5 μm and, in line with animal studies, the presence of NGF and GDNF also increased the percentage of TRPV1 immunoreactive neurons coupled with an increased response to the TRPV1 agonist capsaicin (Anand et al., 2006).

##### Nitric oxide synthase (NOS)

Nitric oxide synthase (NOS) isoforms 1–3 are present in DRG and its product nitric oxide (NO) is involved in nociceptive signaling with evidence supporting analgesic and algesic actions. In particular NOS1 (neuronal NOS) has been shown to be upregulated in DRG neurons in animal models of neuropathic and inflammatory pain (Cury et al., 2011). NOS1 has been described in small-medium sized human DRG neurons and was present in 40–50% of all neurons in the investigated ganglia (Terenghi et al., 1993).

##### Gamma amino butyric acid (GABA)

Gamma amino butyric acid (GABA) and its receptors are the main inhibitors in the nervous system. GABA_A_-receptors are ligand-gated chloride channels whereas GABA_B_ receptors are G-protein coupled receptors. Both are expressed in DRG neurons. Activation of GABA_A_ leads to conformational change in GABA_B_, inhibiting the excitability of neurons via blockade of Ca_V_ channels (Huang et al., 2015; Zhang et al., 2015). Animal models showed that GABA_A_-receptors have a role in the pathophysiology of neuropathic pain (Wang et al., 2017).

Pharmacological evidence for the presence of GABA_A_ receptors in human DRG neurons, has been provided by experiments that completely blocked GABA-induced currents by the GABA_A_ receptor antagonists, bicuculline and picrotoxin. Interestingly, electrophysiological properties of GABA_A_-mediated current were different between human and mouse DRG neurons (Zhang et al., 2015).

Additional evidence for the presence and involvement of GABA_B_ receptors in the excitability of human DRG neurons has been provided from experiments investigating the inhibitory action of a cone-snail venom V_C_1.1. Those experiments showed the expression of GABA_B_ and demonstrated the absence of an inhibitory effect V_C_1.1 when GABA_B_ could not be activated (Castro et al., 2017).

##### Phospholipase β3

Phospholipases (PLC) are present in DRG neurons and participate in pain signaling (Joseph et al., 2007). Phospolipases β, γ, δ, and 𝜀 are part of a magnitude of different signaling pathways with the PLC β3 isoform implicated in nociceptive signaling. This isoform is present in small human DRG neurons and colocalises with CGRP immunoreactive and I-B4 positive neurons (Shi et al., 2008).

## Summary and Conclusion

In summary, only a small population of molecules that have been described to be involved in the function of DRG neurons in laboratory animals have so far been investigated in humans. It is evident from existing studies that expression patterns and functions of molecules in DRG do not perfectly match between human and laboratory animal. Important differences exist between human DRG compared to laboratory animals and careful conducted future studies will be essential to reconcile and validate these to appropriately translate animal data into human context. At the physiological level, the longer peripheral processes and associated soma size of human DRG is likely to account for some of these differences, such as immunoreactivity for neurofilament 200 in all human DRG neurons including those classified as large. Similarly, many molecules characteristic of nociceptors including TRPV1, CGRP and P_2_X_3_ and voltage-gated sodium channels are restricted to small and medium sized neurons in mice but in not in humans where nociception-related proteins (immunohistochemistry) and mRNAs (*in situ* hybridization) are present in neurons of all sizes. Furthermore, ion channel proteins such as TRPV1, Na_V_1.8, Na_V_1.9, and nicotinic receptor subtypes seem to be expressed in larger proportions of human DRG nociceptors compared to mice (Rostock et al., 2017; Zhang et al., 2019). In addition, the separation of nociceptive DRG neurons into NGF- and GDNF-dependent populations might also be questioned in relation to human DRG as the GDNF receptor protein RET is present in neurons that express the receptor for NGF, TrkA (Rostock et al., 2017).

By all means, these discrepancies do not completely invalidate results from animal studies in a human translational context. Indeed, most human DRG neurons show remarkably similar patterns in respect to immunoreactivities for pain-related molecules being detected in smaller sized neurons characteristic for nociceptors. But the diversity across sizes combined with differences in electrophysiological properties (Zhang et al., 2015, 2017, 2019) suggests a more complex array of human DRG neuronal subtypes, that may differ in detecting and conveying nociceptive information when compared to laboratory animals such as rats and mice.

Regardless of species, DRG contain multiple types of neurons and multiple types of other cells including satellite cells and cells associated with immune and vascular function. Dissociation of ganglia and culture of primary sensory neurons is useful to identify neuronal characteristics, but inferences from these studies must recognize that some types of neurons, specifically those with larger size, will likely not survive mechanical isolation and subsequent culture conditions. More importantly, critical issues related to antibody specificity highlight challenges relevant to data collections from both human and animal tissues, including the ability to compare neuronal subpopulations across species.

An emerging and clinically significant area for further investigation is the interaction of neuronal and non-neuronal cells within DRG, and certain neuron-immune cell interactions involved in pain sensitivity have been shown to be consistent in humans and in laboratory animals. Sensory neuron-immune cell interactions are increasingly recognized as important mechanisms that contribute to chronic pain, yet there is surprisingly sparse investigative reporting of cells such as macrophages and satellite cells in human DRG. In the next few decades, researchers will hopefully find increasing opportunities to investigate and validate molecular and cellular characteristics of human DRG tissues. The failure of swathes of clinical trials based on animal model data in the past few decades reinforces the importance of human studies in clinical translation and therapeutic development, especially in very complex conditions such as chronic pain. Early insights from a handful of comparative studies suggest fundamental differences in molecular characteristics of rodent and human DRG nociceptive neurons, as well as other cell types in the DRG, and may provide key pieces of information to select optimal targets and aid more effective drug design strategies.

## Author Contributions

RH planned the manuscript. RH, DM, CB, and ND wrote the manuscript.

## Conflict of Interest Statement

The authors declare that the research was conducted in the absence of any commercial or financial relationships that could be construed as a potential conflict of interest.
