# Supplementary material for: Human Dorsal Root Ganglia
Source: Front Cell Neurosci. 2019 Jun 19;13:271. doi: 10.3389/fncel.2019.00271 (PMC6598622; doi:10.3389/fncel.2019.00271)
Supplement: Supplementary file 1 [file Table_1.docx]

Supplementary Material

**Supplementary table 1**

List of nociceptor-related molecules described in human DRG neurons using immunohistochemistry including information about the antisera used, numbers of specimen and the use of appropriate controls. Specificity control: accepted controls for the specificity of the primary antiserum/antibody were preabsorption with the corresponding antigen, Western blots showing the complete blot with a single band at the correct molecular weight. As not ideal control for specificity of immunoreactivity in human DRG was considered the absence of immunoreactivity in antigen-deficient (knock-out) cells or animals.

Abbreviations: Rb – Rabbit, Ms – mouse, Gt – goat, Ch – chicken, GP – guinea-pig, m – male, f - female

| **Antigen** | **Host, antibody supplier** | **Reference** | **Number DRG, donors, gender** | **Specificity control** |
| --- | --- | --- | --- | --- |
| NaV1.7 | Rb, Alomone | (Li, North et al. 2018) | 6 DRG; 3 donors (2m;1f) | Preabsorption |
| PN1 (TTX-S) Nav1.7 | Rb, not specified | (Coward, Aitken et al. 2001) | n of DRG not specified. (5 m; 4f) | Preabsorption and Western blot |
| SNS/PN3 (TTX resistant voltage-gated sodium channel) Nav1.8 | Rb, (Coward, Plumpton et al. 2000) | (Coward, Plumpton et al. 2000) | n of DRG not specified; 12 donors (12m) | Preabsorption |
| NaN/SNS2 (TTX resistant voltage gated sodium channel) Nav1.9 | Rb, (Coward, Plumpton et al. 2000) | (Coward, Plumpton et al. 2000) | n of DRG not specified; 12 donors (12m) | Preabsorption |
| TRPV1 | GP, Abcam | (Li, North et al. 2018) | 6 DRG; 3 donors (2m;1f) | Preabsorption |
| TRPV1 | Rb, Neuromics | (Chang, Berta et al. 2018) | n of DRG not specified; 15 donors, gender not specified | Preabsorption |
| TRPV1 | Rb, (Valtcheva, Copits et al. 2016) | (Valtcheva, Copits et al. 2016) | Not specified | Absence of staining in TRPV1^-/-^ mice |
| TRPV1 | Rb, Pierce | (Enright, Felix et al. 2016) | n of DRG not specified; 1 female donor; (1f) | No |
| TRPV1 | Rb, Alomone | (Li, Adamek et al. 2015) | Not specified | No |
| TRPV1 | Rb, GlaxoSmithKline | (Anand, Facer et al. 2013) | 4 DRG, 2 donors; (2f) | Preabsorption (Smith, Gunthorpe et al. 2002) |
| TRPV1 | Rb, GlaxoSmithKline | (Anand, Otto et al. 2008) | n of DRG not specified; 6 donors; gender not specified | Preabsorption (Smith, Gunthorpe et al. 2002) |
| TRPV1 | Rb, GlaxoSmithKline | (Facer, Casula et al. 2007) | 8 DRG; 3 donors; (3m) | Preabsorption (Smith, Gunthorpe et al. 2002) |
| TRPV1 | Not specified, R&D (GSK) | (Lauria, Morbin et al. 2006) | n of DRGs not specified; 3 donors; (3f) | Preabsorption |
| TRPV3 | Rb, GSK | (Facer, Casula et al. 2007) | 8 DRG; 3 donors; (3m) | Preabsorption (Smith, Gunthorpe et al. 2002) |
| TRPA1 | Rb GSK | (Anand, Otto et al. 2008) | 8 DRG; 7 donor; (7m) | Preabsorption and Western blot |
| CGRP | Ms, Abcam | (Li, North et al. 2018) | 6 DRGs; 3 donors (2m;1f) | No |
| CGRP | Rb, Hoekfelt Lab | (Shi, Xiang et al. 2012) | n of DRGs not specified; DRG from children with obstetric brachial plexus lesions | Preabsorption in rat tissue |
| CGRP | Ms, Sigma | (Pan, Wu et al. 2012) | n of DRGs not specified. 12 donors (abortion). | No |
| CGRP | Rb, Sigma | (Pan, Wu et al. 2012) | n of DRGs not specified. 12 donors (abortion). | No |
| CGRP | Ch, GenTex | (Patil, Schwab et al. 2010) | Donors not specified; N=4 DRGs | No |
| CGRP | Rb, (Platt et al., J Histochem, Cytochem 1983) | (Shi, Liu et al. 2008) | n of DRGs not specified; DRG from children with obstetric brachial plexus lesions | No |
| CGRP | Rb, Peninsula | (Nordlind, Eriksson et al. 2000) | n DRGs not specified; 3 donors (3m), 3 foetal donors | No |
| CGRP | Rb, (Marti, Gibson et al. 1987) | (Suburo, Gu et al. 1992) | 19 DRGs; 45 donors (DRGs not obtained from all donors) ranging from 7 gestational weeks to adults; gender not specified | Preabsorption |
| α-CGRP | Rb, Peninsula | (Giaid, Gibson et al. 1989) | n of DRGs not specified; 10 donors; gender not specified | Preabsorption |
| CGRP | Rb, (Gibson, Polak et al. 1984) | (Marti, Gibson et al. 1987) | n of DRGs not specified; 152 foetal donors; gender not specified | Preabsorption |
| SP | Rb, Peninsula | (Nordlind, Eriksson et al. 2000) | n DRGs not specified; 3 donors (3m), 3 foetal donors | No |
| Substance P | Rb, (Marti, Gibson et al. 1987) | (Suburo, Gu et al. 1992) | 19 DRGs; 45 donors (DRGs not obtained from all donors) ranging from 7 gestational weeks to adults; gender not specified | Preabsorption |
| Substance P | Rb, Incstar | (Nagao, Oka et al. 1994) | n of DRGs not specified; 6 donors; (6m) | No |
| Substance P | Rb, (Gibson, Polak et al. 1981) | (Marti, Gibson et al. 1987) | n of DRGs not specified; 152 foetuses; gender not specified | Preabsorption |
| Galanin | Rb, (Marti, Gibson et al. 1987) | (Suburo, Gu et al. 1992) | 19 DRGs; 45 donors (DRGs not obtained from all donors) ranging from 7 gestational weeks to adults; gender not specified | Preabsorption |
| Galanin | Rb, (Ch'ng, Christofides et al. 1985) | (Marti, Gibson et al. 1987) | n of DRGs not specified; 152 foetuses; gender not specified | Preabsorption |
| Somatostatin | Rb, Incstar | (Nagao, Oka et al. 1994) | n of DRGs not specified; 6 donors; (6m) | No |
| Somatostatin | Rb, (McGregor, Gibson et al. 1984) | (Marti, Gibson et al. 1987) | n of DRGs not specified; 152 foetal donors; gender not specified | Preabsorption |
| Somatostatin | Rb, SS14 | (Charnay, Chayvialle et al. 1987) | n of DRGs not specified, 3 embryonal and 29 foetal donors (9-36 weeks); gender not specified | Preabsorption |
| Somatostatin | Rb, SS28_1-12_ | (Charnay, Chayvialle et al. 1987) | n of DRGs not specified, 3 embryonal and 29 foetal donors (9-36 weeks); gender not specified | Preabsorption |
| Somatostatin | Rb, SS28_1-12_ | (Charnay, Chayvialle et al. 1987) | n of DRGs not specified, 3 embryonal and 29 foetal donors (9-36 weeks); gender not specified | Preabsorption |
| Somatostatin 2A receptor (Sst2A) | Clone UMB-1 | (Shi, Xiang et al. 2014) | n of DRGs not indicated; 1 donor; (1f) | No |
| Endothelin-1 | Rb, made | (Giaid, Gibson et al. 1989) | n of DRGs not specified; 10 donors; gender not specified | Preabsorption |
| Isolectin B4 (IB4) | Vector | (Shi, Xiang et al. 2012) | n of DRGs not specified; DRG from children with obstetric brachial plexus lesions | No |
| Isolectin B4 (IB4, biotinylated) | Sigma | (Pan, Wu et al. 2012) | n of DRGs not specified. 12 donors | No |
| Isolectin B4 (IB4) | Vector Lab | (Shi, Liu et al. 2008) | n of DRGs, donors and gender not specified | No |
| P2X3 | Rb, Abcam | (Pan, Wu et al. 2012) | n of DRGs not specified. 12 donors | Preabsorption and Western blot |
| P2X3 | Rb, Roche | (Yiangou, Facer et al. 2000) | 5 DRG; 5 donors (post mortem); gender not specified | Preabsorption and Western blot |
| GAP-43 | Ms, Sigma | (Anand, Otto et al. 2008) | n of DRGs not specified; 6 donors; gender not specified | No |
| AngII | Ms | (Patil, Schwab et al. 2010) | 4 DRGs; donors and gender not specified | Similar staining antisera in rat tissue |
| AT2R | Gt, Santa Cruz | (Anand, Facer et al. 2013) | 4 DRGs; 2 donors; (2w) | Preabsorption |
| PLCβ3 | GP, (Nomura, Fukaya et al. 2007) | (Shi, Liu et al. 2008) | n of DRGs not specified; DRG from children with obstetric brachial plexus lesions | Preabsorption |
| GLP-1R | Not specified, Abcam | (Anand, Yiangou et al. 2018) | n of DRGs not specified; 3 donors; gender not specified | Preabsorption |
| NOS | NADPH diaphorase | (Nagano, Shapshak et al. 1996) | n of DRGs not indicated; 5 donors; gender not specified | Absence of substrate |
| TrkA | Not specified | (Yiangou, Facer et al. 2000, Rostock, Schrenk-Siemens et al. 2017) | 5 DRG, 5 donors; (1m, 4f) | No |
| TrkA | Ms, Genentech | (Yiangou, Facer et al. 2000) | 5 DRG; 5 donors obtained post mortem; gender not specified | No |
| TrkA | Rb, Genentech | (Coward, Aitken et al. 2001) | n of DRG not specified; 12 donors; (12m) | No |
| TrkA | Rb, St Cruz | (Vega, Vazquez et al. 1994) | 11 DRG, 5 donors; gender not specified | No |
| TrkB | Not specified | (Rostock, Schrenk-Siemens et al. 2017) | 5 DRG, 5 donors; (1m, 4f) | No |
| TrkB | Ms, Abgent | (Schonemann, Muench et al. 2012) | n of DRGs not specified; donors n not specified | No |
| TrkC | Not specified | (Rostock, Schrenk-Siemens et al. 2017) | 5 DRG, 5 donors; (1m, 4f) | No |
| Low affinity NGF receptor | Ms, ME20.4, (Ross, Grob et al. 1984) | (Suburo, Gu et al. 1992) | 19 DRGs; 45 donors (DRGs not obtained from all donors) ranging from 7 gestational weeks to adults; gender not specified | No |
| GDNF | Rb, St Cruz | (Bar, Saldanha et al. 1998) | 7 DRGs, n of donors not specified | Preabsorption |
| RET | Rb, not specified | (Bar, Saldanha et al. 1998) | 7 DRGs, n of donors not specified | Preabsorption |

Anand, U., P. Facer, Y. Yiangou, M. Sinisi, M. Fox, T. McCarthy, C. Bountra, Y. E. Korchev and P. Anand (2013). "Angiotensin II type 2 receptor (AT2 R) localization and antagonist-mediated inhibition of capsaicin responses and neurite outgrowth in human and rat sensory neurons." Eur J Pain **17**(7): 1012-1026.

Anand, U., W. R. Otto, C. Bountra, I. Chessell, M. Sinisi, R. Birch and P. Anand (2008). "Cytosine arabinoside affects the heat and capsaicin receptor TRPV1 localisation and sensitivity in human sensory neurons." J Neurooncol **89**(1): 1-7.

Anand, U., W. R. Otto, P. Facer, N. Zebda, I. Selmer, M. J. Gunthorpe, I. P. Chessell, M. Sinisi, R. Birch and P. Anand (2008). "TRPA1 receptor localisation in the human peripheral nervous system and functional studies in cultured human and rat sensory neurons." Neurosci Lett **438**(2): 221-227.

Anand, U., Y. Yiangou, A. Akbar, T. Quick, A. MacQuillan, M. Fox, M. Sinisi, Y. E. Korchev, B. Jones, S. R. Bloom and P. Anand (2018). "Glucagon-like peptide 1 receptor (GLP-1R) expression by nerve fibres in inflammatory bowel disease and functional effects in cultured neurons." PLoS One **13**(5): e0198024.

Bar, K. J., G. J. Saldanha, A. J. Kennedy, P. Facer, R. Birch, T. Carlstedt and P. Anand (1998). "GDNF and its receptor component Ret in injured human nerves and dorsal root ganglia." Neuroreport **9**(1): 43-47.

Ch'ng, J. L., N. D. Christofides, P. Anand, S. J. Gibson, Y. S. Allen, H. C. Su, K. Tatemoto, J. F. Morrison, J. M. Polak and S. R. Bloom (1985). "Distribution of galanin immunoreactivity in the central nervous system and the responses of galanin-containing neuronal pathways to injury." Neuroscience **16**(2): 343-354.

Chang, W., T. Berta, Y. H. Kim, S. Lee, S. Y. Lee and R. R. Ji (2018). "Expression and Role of Voltage-Gated Sodium Channels in Human Dorsal Root Ganglion Neurons with Special Focus on Nav1.7, Species Differences, and Regulation by Paclitaxel." Neurosci Bull **34**(1): 4-12.

Charnay, Y., J. A. Chayvialle, L. Pradayrol, R. Bouvier, C. Paulin and P. M. Dubois (1987). "Ontogeny of somatostatin-like immunoreactivity in the human fetus and infant spinal cord." Brain Res **433**(1): 63-73.

Coward, K., A. Aitken, A. Powell, C. Plumpton, R. Birch, S. Tate, C. Bountra and P. Anand (2001). "Plasticity of TTX-sensitive sodium channels PN1 and brain III in injured human nerves." Neuroreport **12**(3): 495-500.

Coward, K., C. Plumpton, P. Facer, R. Birch, T. Carlstedt, S. Tate, C. Bountra and P. Anand (2000). "Immunolocalization of SNS/PN3 and NaN/SNS2 sodium channels in human pain states." Pain **85**(1-2): 41-50.

Enright, H. A., S. H. Felix, N. O. Fischer, E. V. Mukerjee, D. Soscia, M. McNerney, K. Kulp, J. Zhang, G. Page, P. Miller, A. Ghetti, E. K. Wheeler and S. Pannu (2016). "Long-term non-invasive interrogation of human dorsal root ganglion neuronal cultures on an integrated microfluidic multielectrode array platform." Analyst **141**(18): 5346-5357.

Facer, P., M. A. Casula, G. D. Smith, C. D. Benham, I. P. Chessell, C. Bountra, M. Sinisi, R. Birch and P. Anand (2007). "Differential expression of the capsaicin receptor TRPV1 and related novel receptors TRPV3, TRPV4 and TRPM8 in normal human tissues and changes in traumatic and diabetic neuropathy." BMC Neurol **7**: 11.

Giaid, A., S. J. Gibson, B. N. Ibrahim, S. Legon, S. R. Bloom, M. Yanagisawa, T. Masaki, I. M. Varndell and J. M. Polak (1989). "Endothelin 1, an endothelium-derived peptide, is expressed in neurons of the human spinal cord and dorsal root ganglia." Proc Natl Acad Sci U S A **86**(19): 7634-7638.

Gibson, S. J., J. M. Polak, S. R. Bloom, I. M. Sabate, P. M. Mulderry, M. A. Ghatei, G. P. McGregor, J. F. Morrison, J. S. Kelly, R. M. Evans and et al. (1984). "Calcitonin gene-related peptide immunoreactivity in the spinal cord of man and of eight other species." J Neurosci **4**(12): 3101-3111.

Gibson, S. J., J. M. Polak, S. R. Bloom and P. D. Wall (1981). "The distribution of nine peptides in rat spinal cord with special emphasis on the substantia gelatinosa and on the area around the central canal (lamina X)." J Comp Neurol **201**(1): 65-79.

Lauria, G., M. Morbin, R. Lombardi, R. Capobianco, F. Camozzi, D. Pareyson, M. Manconi and P. Geppetti (2006). "Expression of capsaicin receptor immunoreactivity in human peripheral nervous system and in painful neuropathies." J Peripher Nerv Syst **11**(3): 262-271.

Li, Y., P. Adamek, H. Zhang, C. E. Tatsui, L. D. Rhines, P. Mrozkova, Q. Li, A. K. Kosturakis, R. M. Cassidy, D. S. Harrison, J. P. Cata, K. Sapire, H. Zhang, R. M. Kennamer-Chapman, A. B. Jawad, A. Ghetti, J. Yan, J. Palecek and P. M. Dougherty (2015). "The Cancer Chemotherapeutic Paclitaxel Increases Human and Rodent Sensory Neuron Responses to TRPV1 by Activation of TLR4." J Neurosci **35**(39): 13487-13500.

Li, Y., R. Y. North, L. D. Rhines, C. E. Tatsui, G. Rao, D. D. Edwards, R. M. Cassidy, D. S. Harrison, C. A. Johansson, H. Zhang and P. M. Dougherty (2018). "DRG Voltage-Gated Sodium Channel 1.7 Is Upregulated in Paclitaxel-Induced Neuropathy in Rats and in Humans with Neuropathic Pain." J Neurosci **38**(5): 1124-1136.

Marti, E., S. J. Gibson, J. M. Polak, P. Facer, D. R. Springall, G. Van Aswegen, M. Aitchison and M. Koltzenburg (1987). "Ontogeny of peptide- and amine-containing neurones in motor, sensory, and autonomic regions of rat and human spinal cord, dorsal root ganglia, and rat skin." J Comp Neurol **266**(3): 332-359.

McGregor, G. P., S. J. Gibson, I. M. Sabate, M. A. Blank, N. D. Christofides, P. D. Wall, J. M. Polak and S. R. Bloom (1984). "Effect of peripheral nerve section and nerve crush on spinal cord neuropeptides in the rat; increased VIP and PHI in the dorsal horn." Neuroscience **13**(1): 207-216.

Nagano, I., P. Shapshak, M. Yoshioka, K. Xin, S. Nakamura and W. G. Bradley (1996). "Increased NADPH-diaphorase reactivity and cytokine expression in dorsal root ganglia in acquired immunodeficiency syndrome." J Neurol Sci **136**(1-2): 117-128.

Nagao, M., N. Oka, H. Kamo, I. Akiguchi and J. Kimura (1994). "Differential localization of lectin binding sites and neuropeptides in human dorsal root ganglia." Histochemistry **102**(4): 279-286.

Nomura, S., M. Fukaya, T. Tsujioka, D. Wu and M. Watanabe (2007). "Phospholipase Cbeta3 is distributed in both somatodendritic and axonal compartments and localized around perisynapse and smooth endoplasmic reticulum in mouse Purkinje cell subsets." Eur J Neurosci **25**(3): 659-672.

Nordlind, K., L. Eriksson, A. Seiger and M. Bakhiet (2000). "Expression of interleukin-6 in human dorsal root ganglion cells." Neurosci Lett **280**(2): 139-142.

Pan, A., H. Wu, M. Li, D. Lu, X. He, X. Yi, X. X. Yan and Z. Li (2012). "Prenatal expression of purinergic receptor P2X3 in human dorsal root ganglion." Purinergic Signal **8**(2): 245-254.

Patil, J., A. Schwab, J. Nussberger, T. Schaffner, J. M. Saavedra and H. Imboden (2010). "Intraneuronal angiotensinergic system in rat and human dorsal root ganglia." Regul Pept **162**(1-3): 90-98.

Ross, A. H., P. Grob, M. Bothwell, D. E. Elder, C. S. Ernst, N. Marano, B. F. Ghrist, C. C. Slemp, M. Herlyn, B. Atkinson and et al. (1984). "Characterization of nerve growth factor receptor in neural crest tumors using monoclonal antibodies." Proc Natl Acad Sci U S A **81**(21): 6681-6685.

Rostock, C., K. Schrenk-Siemens, J. Pohle and J. Siemens (2017). "Human vs. Mouse Nociceptors - Similarities and Differences." Neuroscience.

Schonemann, M. D., M. O. Muench, M. K. Tee, W. L. Miller and S. H. Mellon (2012). "Expression of P450c17 in the human fetal nervous system." Endocrinology **153**(5): 2494-2505.

Shi, T. J., S. X. Liu, H. Hammarberg, M. Watanabe, Z. Q. Xu and T. Hokfelt (2008). "Phospholipase C{beta}3 in mouse and human dorsal root ganglia and spinal cord is a possible target for treatment of neuropathic pain." Proc Natl Acad Sci U S A **105**(50): 20004-20008.

Shi, T. J., Q. Xiang, M. D. Zhang, S. Barde, Y. Kai-Larsen, K. Fried, A. Josephson, L. Gluck, S. M. Deyev, A. V. Zvyagin, S. Schulz and T. Hokfelt (2014). "Somatostatin and its 2A receptor in dorsal root ganglia and dorsal horn of mouse and human: expression, trafficking and possible role in pain." Mol Pain **10**: 12.

Shi, T. J., Q. Xiang, M. D. Zhang, G. Tortoriello, H. Hammarberg, J. Mulder, K. Fried, L. Wagner, A. Josephson, M. Uhlen, T. Harkany and T. Hokfelt (2012). "Secretagogin is expressed in sensory CGRP neurons and in spinal cord of mouse and complements other calcium-binding proteins, with a note on rat and human." Mol Pain **8**: 80.

Smith, G. D., M. J. Gunthorpe, R. E. Kelsell, P. D. Hayes, P. Reilly, P. Facer, J. E. Wright, J. C. Jerman, J. P. Walhin, L. Ooi, J. Egerton, K. J. Charles, D. Smart, A. D. Randall, P. Anand and J. B. Davis (2002). "TRPV3 is a temperature-sensitive vanilloid receptor-like protein." Nature **418**(6894): 186-190.

Suburo, A. M., X. H. Gu, G. Moscoso, A. Ross, G. Terenghi and J. M. Polak (1992). "Developmental pattern and distribution of nerve growth factor low-affinity receptor immunoreactivity in human spinal cord and dorsal root ganglia: comparison with synaptophysin, neurofilament and neuropeptide immunoreactivities." Neuroscience **50**(2): 467-482.

Valtcheva, M. V., B. A. Copits, S. Davidson, T. D. Sheahan, M. Y. Pullen, J. G. McCall, K. Dikranian and R. W. t. Gereau (2016). "Surgical extraction of human dorsal root ganglia from organ donors and preparation of primary sensory neuron cultures." Nat Protoc **11**(10): 1877-1888.

Vega, J. A., E. Vazquez, F. J. Naves, M. E. Del Valle, B. Calzada and J. J. Represa (1994). "Immunohistochemical localization of the high-affinity NGF receptor (gp140-trkA) in the adult human dorsal root and sympathetic ganglia and in the nerves and sensory corpuscles supplying digital skin." Anat Rec **240**(4): 579-588.

Yiangou, Y., P. Facer, R. Birch, L. Sangameswaran, R. Eglen and P. Anand (2000). "P2X3 receptor in injured human sensory neurons." Neuroreport **11**(5): 993-996.
